# Teachers’ perceptions of physical activity in children and young people with type 1 diabetes: a qualitative systematic review protocol

**DOI:** 10.1186/s13643-026-03279-7

**Published:** 2026-08-08

**Authors:** Monika Jakiel-Rusin, Julian Hamilton-Shield, Robert C. Andrews, Vincent Singh

**Affiliations:** 1 NIHR Bristol Biomedical Research Centre, Oakfield House, Oakfield Grove, Bristol, BS8 2BN UK; 2 Bristol Royal Hospital for Children, BFT, Upper Maudlin Street, Bristol, BS2 8BJ UK; 3 University of Bristol, Senate House, Tyndall Avenue, Bristol, BS8 1TH UK; 4 University of Exeter Medical School, St Luke’s Campus, Heavitree Road, Exeter, EX1 2LU UK; 5 College of Health, Science and Society, University of the West of England, Bristol, BS161DD UK

**Keywords:** Type 1 diabetes, Physical activity, Teachers, School health, Children and young people, Qualitative evidence synthesis

## Abstract

**Background:**

Type 1 diabetes is a lifelong condition usually diagnosed in childhood. It requires daily management to control blood glucose and avoid complications. Physical activity brings important physical and emotional benefits, but children with the condition are often less active than their peers. Schools have a key role in encouraging active lifestyles, yet managing diabetes around physical activity can be difficult and requires specific knowledge. Teachers are central to enabling participation, but many feel unprepared owing to limited training, low confidence, and persistent misconceptions. Exploring their views is vital for improving school-based support. This review aims to assess and summarise qualitative research on teachers’ knowledge, attitudes, and experiences in supporting children with type 1 diabetes in physical activity, to guide future training, policy, and practice.

**Methods:**

This review will follow the Joanna Briggs Institute methodology for qualitative evidence synthesis, using meta-aggregation. A comprehensive search will be conducted across multiple databases: MEDLINE, Embase, CINAHL, PsycINFO, ERIC, ProQuest, and TRIP Database. The SPIDER tool will guide the search strategy and inclusion criteria. All records will be independently screened and appraised by at least two reviewers using the Joanna Briggs Institute critical appraisal tool, with discrepancies resolved through discussion. NVivo 15 qualitative data analysis software will be used to support data management, organisation, and analysis. Confidence in the findings will be assessed using the GRADE-CERQual approach. The review will follow PRISMA 2020 guidelines to ensure transparency and methodological rigour.

**Discussion:**

This review addresses a key evidence gap and considers practical and contextual challenges across school settings. Practical and contextual challenges, including variations in study reporting, educational systems, and school-level policies, will be considered. A multidisciplinary review team will ensure methodological rigour and support nuanced interpretation. While the exclusion of non-English studies may introduce language bias and systemic factors influencing teacher perspectives may not always be fully captured, the review adopts a transparent and robust methodology. The findings will inform tailored training, school-based strategies, and policy recommendations to improve teacher confidence and enable safer, more inclusive physical activity participation.

**Systematic review registration:**

PROSPERO 2025 CRD420251071101

**Supplementary Information:**

The online version contains supplementary material available at https://doi.org/10.1186/s13643-026-03279-7.

## Main body

### Background

Type 1 diabetes (T1D) is a chronic autoimmune condition characterised by insufficient insulin production, resulting in lifelong dependency on exogenous insulin and vigilant blood glucose management. Although T1D can develop at any age, it is mostly diagnosed in childhood and adolescence, particularly in those under 15 years of age. An estimated 108,300 children under 15 years of age were diagnosed globally in 2021, with this number increasing to 149,500 when the age range is extended to under 20 years [1], accounting for 40% of new diagnoses [2]. Globally, the prevalence of T1D among children and young people is increasing, with the incidence increasing by 3–4% annually in many regions [3]. In the UK, approximately 1 in every 1000 children is affected—one of the highest prevalence rates in Western Europe [4].

This early onset is particularly notable because affected children and young people (CYP) must manage complex, lifelong conditions during critical developmental stages, which impacts their physical, emotional, and social well-being. These findings underscore the importance of tailored healthcare support and education to help young people, and their families manage their condition effectively throughout key stages of growth and development. If poorly managed, T1D can lead to severe long-term complications, including cardiovascular disease, renal failure, and retinopathy [5].

Regular physical activity (PA) offers wide-ranging health benefits for CYP with T1D, including enhanced glycaemic control, improved insulin sensitivity, better cardiovascular fitness, and mental well-being [6, 7]. There is also evidence that PA can help extend the “honeymoon period” following diagnosis, a time shortly after diagnosis when the body still produces some insulin and blood sugar levels are easier to manage [8]. Despite such benefits and the inclusion of PA in global guidelines [9], approximately 80% of children and CYP fail to meet the recommended levels of at least 60 min of moderate-to-vigorous PA per day. CYP with T1D are consistently less active than their peers are [10]. PA levels decline further during the transition to secondary school, particularly among young girls, due to factors such as reduced opportunities for structured activity, increased academic pressure and a lack of tailored support or encouragement from schools, families, and peers to remain active [11, 12].

Schools are critical environments for promoting PA through physical education (PE), extracurricular opportunities, and active break times [9]. However, for children with T1D, PA at school poses unique management challenges. Exercise-induced glycaemic variability, including risks of hypo- and hyperglycaemia, requires tailored strategies, including blood glucose monitoring, insulin adjustment, and carbohydrate intake [3]. Effective diabetes management during school hours is essential to safeguard health and facilitate participation. Additional pressures such as school trips and sport events often increase the burden of planning and require a deeper understanding from staff [13].

Teachers play a pivotal role in either enabling or limiting PA participation [14]. However, studies consistently show that educators feel underprepared to support CYP with T1D due to limited training, a lack of confidence, and persistent misconceptions [15, 16]. In some cases, this leads to unnecessary restrictions based on fear rather than clinical guidance. A growing body of international research—including studies from Europe, the Middle East, Asia, and the UK—highlights inadequate teacher knowledge, poor communication between schools and families, and a lack of systemic support [17–23]. There have been increasing calls for structured diabetes education to be integrated into initial teacher training and continuing professional development [24, 25].

Several strategies have been proposed to improve support for children with T1D in schools, including individual healthcare plans (IHPs), clear emergency protocols, and effective communication between schools, families, and healthcare professionals [26, 27]. In the UK, these approaches align with the Department for Education’s statutory guidance for supporting pupils with medical conditions [28]. However, successful implementation requires teachers to be adequately prepared and supported. Although structured diabetes education has been shown to improve teachers’ confidence and diabetes-specific knowledge, uptake remains inconsistent, and many educators continue to report uncertainty in supporting PA safely [15, 29].

#### Identified gap and need for a systematic review

A preliminary literature search identified a significant gap in synthesised research exploring teachers’ perceptions of PA for CYPs with T1D. Numerous individual studies have reported the challenges faced by school staff when supporting students with type 1 diabetes [17, 21, 30–32]. However, no systematic review to date has examined PE teachers' perspectives, knowledge, and experiences through a qualitative lens. This gap limits the ability of policymakers, schools, and healthcare providers to design informed, evidence-based training and interventions.

Given teachers’ central role in facilitating safe and inclusive PA, synthesising qualitative evidence on their perspectives will provide important insights into the barriers and facilitators influencing practice. These findings will inform practical and policy-level responses. This review aligns with James Lind Alliance’s priorities for improving support for people living with T1D [33] and addresses NHS ambitions around prevention, long-term condition management, and integrated care [34]. In particular, this work responds to priorities such as understanding what makes self-management successful, improving hypoglycaemia awareness, and identifying effective education approaches—all of which are influenced by the support that CYP with T1D receive in school settings. Enhancing teacher preparedness could contribute to better diabetes control, reduce the burden on the healthcare system, and improve quality of life and educational outcomes for CYP living with T1D.

Aim:

To evaluate and synthesise qualitative evidence on teachers’ knowledge, perceptions, and experiences of supporting CYP with T1D in PA settings to inform future training and support strategies for educators.

Objectives:

1. To analyse existing research on what teachers know and understand about supporting CYP with T1D in relation to PA and exercise.
2. To identify barriers and facilitators that influence teachers’ ability to support CYP with T1D in PA settings.
3. To synthesise the available evidence to inform research priorities and guide the development of targeted training and support interventions for school staff.

### Method and design

This qualitative SR aims to synthesise evidence on teachers’ perceptions of PA in CYP with T1D. An SR methodology was selected to ensure a transparent, rigorous synthesis of qualitative data, integrating diverse perspectives across studies that meet predefined inclusion criteria [35–37]. The process will include defining eligibility criteria, conducting a comprehensive search, screening and selecting studies, critically appraising included studies, extracting data, and synthesising findings. Each stage is guided by established frameworks to ensure clarity and reproducibility.

The review will follow the Joanna Briggs Institute (JBI) Manual for Evidence Synthesis [38]. The JBI Manual provides structured guidance for study selection, quality appraisal, data extraction, and synthesis. A PRISMA flowchart is used to document the selection process [39].

This review follows the JBI methodology for qualitative evidence synthesis, using meta-aggregation as the synthesis approach [38]. JBI meta-aggregation is designed to preserve the meaning of the original findings while producing synthesised statements that are directly applicable to policy and practice. NVivo 15 software will be used to support the organisation and coding of qualitative data throughout the JBI meta-aggregation process, ensuring a transparent, systematic and reproducible synthesis.

To guide the search strategy and define the inclusion criteria, the SPIDER tool (Sample, Phenomenon of Interest, Design, Evaluation, Research Type) will be used. SPIDER is particularly suited to qualitative and mixed-methods reviews, enabling the inclusion of rich, contextual data [40, 41]. This also helps reduce selective outcome reporting and enhances methodological rigour [42].

Finally, the protocol will be registered with PROSPERO to promote transparency, avoid duplication, and support quality and consistency in the review process [42].

#### Search strategy

Search strategies for systematic reviews should ideally be developed with input from information specialists to ensure methodological rigour [38]. In line with the *Systematic Reviews – Policy on Knowledge and Library Service (KLS) Support* at University Hospitals Bristol and Weston NHS Foundation Trust, support from a professional librarian is agreed upon to codevelop and refine the search strategy. This is recognised as a time-intensive process, and their contribution will be acknowledged through co-authorship in any resulting publication, as per institutional policy.

A comprehensive JBI-guided search strategy is implemented in three phases [38]. It begins with an initial scoping search in Ovid MEDLINE, which is structured via the SPIDER framework and informed by four seed papers (see Tables 1 and 2). The strategy combines MeSH and free-text terms with Boolean and proximity operators (e.g. ADJn) to ensure a balance between sensitivity and specificity. Titles, abstracts, and indexing terms from relevant studies are reviewed iteratively to refine the search strategy. Once the MEDLINE search is optimised, it is adapted for CINAHL and other relevant databases, considering database-specific syntax and features.

**Table 1:** Logic grid mapping the review question via the SPIDER framework

**S**ample/setting	Teachers, schools
**P**henomenon of** i**nterest	Perceptions, knowledge, experiences Physical activity, exercise Children, young people Type 1 diabetes
**D**esign	Qualitative study designs (e.g. interviews, focus groups, ethnographic studies)
**E**valuation (outcomes)	Subjective experiences, views, beliefs, and reported barriers/enablers
**R**esearch type	Qualitative (and possibly mixed methods with qualitative findings reported)

**Table 2:** Preliminary search strategy in Ovid Medline

Ovid MEDLINE(R) ALL <1946 to June 09, 2025> 1(school* or school-based or nurser* or kindergarten or high-school* or middle-school* or elementary-school* or "educational institution*" or "educational setting*" or education* or teacher* or educator* or coach* or "school* staff*" or "physical education teacher*" or "PE teacher*" or "general education teacher*").ti, ab, kw.1168360 2(school* adj3 (policy or policies or training)).ti, ab, kw.7758 3("after school*" or afterschool* or "before school*" or "out of school*" or extracurricular* or "outside school hours" or "school camp*" or "sport event*" or "sport day*" or "sport* club*" or "school club*" or "holiday* club*").ti, ab, kw.8671 4educational personnel/or school teachers/4046 5schools/or schools, nursery/57903 6or/1–51174127 7(perception* or perspective* or perceive* or opinion* or knowledg* or experience* or attitude* or view* or understand* or practice* or believe* or belief* or confiden* or awareness).ti, ab, kw.7022748 8perception/or thinking/64612 9attitude/or health knowledge, attitudes, practice/191884 10or/7–97078160 11(PE or "physical education" or sport* or gym*).ti, ab, kw.213526 12(physical* adj3 (activit* or exercise* or education* or movement* or endurance or training)).ti, ab, kw.221217 13(fitness or exercis* or sport* or aerobic* or walk* or athletic* or performance* or run* or baseball or basketball or cycling or football or soccer or swim* or tennis or squash or badminton or hiking or rugby or athletics).ti, ab, kw.2626437 14(scouts or guides or brownies or cubs or explorers or "Duke of Edinburgh" or camping or hiking).ti, ab, kw.27204 15"Physical Education and Training"/14470 16exp Exercise/or exp sports/385992 17or/11–162903055 18(Paediatric* or pediatric* or child* or adolescen* or CYP or preschool* or teen* or prepubescent* or juvenile* or young or youth or toddler* or pupil* or student* or schoolchild* or parent* or famil* or caregiver* or care-giver*).ti, ab, kw.4589638 19adolescent/or athletes/or students/or child/or child, preschool/3609022 20Pediatrics/59955 21or/18–206342326 22(("type 1" or "type one" or juvenile or "juvenile-onset" or "Insulin-dependent") adj diabet*).ti, ab, kw.85783 23(T1D or "diabetes mellitus type 1").ti, ab, kw.14396 24diabetes mellitus, type 1/or hyperglycemia/or hypoglycemia/145028 25or/22–24175990 26(barrier* or hurdle* or obstacle* or imped* or avoid* or fear* or challenge* or factor*).ti, ab, kw.6653110 27(support* or enable* or help* or encourage* or facilitat* or determin* or participat* or engage* or interven* or motivat* or percept* or attitude* or promot* or improv*).ti, ab, kw.13202535 28Fear/41728 29Motivation/85489 30Health Promotion/85520 31or/26–3016336194 32(qualitative or interview* or themes or "ethnographic stud*" or "mixed method*" or "systematic review" or "meta synthes*" or meta-synthes* or Literature review* or questionnaire* or "focus group*" or "thematic analysis" or "case stud*" or "ethnograph*" or "observational stud*" or "grounded theory" or "narrative inquiry" or "cross sectional" or "cross-sectional" or "analytic stud*" or phenomenolog*).ti, ab, kw.2681593 33Qualitative Research/105084 34Interviews as Topic/75115 35or/32–342697418 366 or 216882399 3710 or 358414386 386 and 17 and 21 and 25 and 31 and 37339 3917 and 25 and 31 and 36 and 371462 4038 or 391462 41limit 40 to yr="2015-2025"808	

To benchmark coverage, the Trip Database is used, and the finalised strategy is reviewed by the academic librarian. Supplementary search techniques include reference list screening and backwards and forward citation tracking, guided by the TARCiS framework, which is particularly suitable for complex qualitative review questions [43]. The complete search strategies for all databases will be provided as supplementary material accompanying the final systematic review.

#### Databases and sources

Databases were selected on the basis of their relevance to health sciences, education, and psychology, reflecting the interdisciplinary nature of the review topic. This approach increases the likelihood of identifying studies relevant to the perceptions of teachers regarding PA in CYP with T1D.

These include:

- Biomedical and health sciences databases: MEDLINE, Embase
- Allied health and nursing literature: CINAHL
- Psychology and behavioural sciences: APA PsycINFO
- Clinical research and guidelines: TRIP Database
- Broader academic sources and education: ERIC
- Databases for Theses and Dissertations – ProQuest

#### Supplementary search methods

Additional resources will be searched through the following:

- Reference list screening of all selected studies for critical appraisal.
- Citation searching by following the TARCiS framework, using SCOPUS citation indices to identify both forward and backwards citations of included studies.
- Experts in the field, including Professor Rob Andrews and Dr Julian Hamilton-Shield, have been consulted regarding the inclusion of grey literature. On the basis of their extensive experience and understanding of the evidence base, they agreed that searching grey literature is unlikely to add significant value to the review findings. Given the time-intensive nature of grey literature searches and the limited likelihood of identifying relevant unpublished studies, it has been agreed that this step is not necessary for this review.

#### Peer review of search strategies

The search strategy will be reviewed using the Peer Review of Electronic Search Strategies (PRESS) checklist [44] and refined by a professional librarian. Searches are updated every six months [35, 38]. Any changes will be documented in the final review or appendix. Search alerts and saved searches support updates, with a final search conducted before manuscript submission.

#### Inclusion and exclusion criteria

The SPIDER framework will guide the development of clear inclusion and exclusion criteria. Additionally, we will consider the publication year and language of the studies. This will provide a structured checklist for study selection [38].

Inclusion criteria:

Studies will focus on the following:

- S: Teachers, teaching assistants, PE staff, coaches or school personnel supporting PA.
- PIs: Perceptions, experiences, knowledge, or attitudes toward supporting PA for CYP with T1D.
- D: Qualitative designs (e.g. interviews, focus groups, ethnography, case studies); mixed methods studies will be included where qualitative findings can be clearly identified and extracted separately. Where qualitative and quantitative findings are closely integrated, studies will be included only if the qualitative findings can be meaningfully distinguished to allow independent qualitative synthesis in accordance with JBI methodology.
- E: Barriers, enablers, or factors influencing PA support in schools.
- R: Original qualitative studies or qualitative elements of mixed-methods research.
- Date: Studies published from 1 January 2015 to the final search date will be included to provide a comprehensive view of how teachers’ perceptions of PA in children and young people with T1D have evolved over time. This extended timeframe captures key developments in diabetes care, educational policy, and PA guidelines. Including older studies allows for contextual insights while ensuring their relevance to current practice. Outdated or no longer applicable studies will be excluded during screening.
- Language: Abstracts in English will be screened, and full texts will be sought when feasible.

Exclusion criteria:

- Quantitative-only studies or studies without extractable qualitative data.
- Research not involving school staff or not addressing T1D or physical activity.
- Full texts published in languages other than English.

#### Study selection process

Study selection will follow the guidance outlined in the JBI Manual for Evidence Synthesis [38]. Papers will be chosen on the basis of their relevance to the review question, using the inclusion and exclusion criteria outlined in Sect. 3.1.4. Eligible studies will focus on qualitative or mixed-methods research exploring school staff members’ knowledge, perceptions, experiences, or attitudes towards PA in CYP with T1D.

Eligibility is determined through a two-stage process:

1. Title and abstract screening to identify relevant studies.
2. A full-text review will performed to confirm eligibility.

Studies meeting the predefined inclusion criteria will be included in the synthesis. The review will follow the JBI meta-aggregation methodology, which seeks to synthesise findings while preserving the meaning of the original qualitative data.

#### Sources

Table 3 outlines the structured study selection process, highlighting key steps and strategies to minimise bias and enhance rigour. It incorporates established tools, frameworks, and dual independent reviews with interrater reliability checks to ensure consistency and transparency.

**Table 3:** A structured table outlining the study selection process to reduce bias

Stage	Description	Measures to reduce bias
Identification	All references retrieved from the final search strategy will be imported into a citation manager (e.g. Mendeley or EndNote), and duplicates will be removed	Use of a single reference library for systematic citation management. Deduplication supported by citation software (e.g. Rayyan or Covidence)
Screening	Titles and abstracts will be screened to determine whether full-text assessment is needed	All records independently screened by two reviewers via Rayyan. Discrepancies discussed in regular meetings with the wider review team
Eligibility	Full texts of potentially relevant studies will be assessed against predefined inclusion and exclusion criteria	Each study screened by two reviewers guided by the SPIDER framework. Discrepancies resolved via discussion in regular meetings with the wider review team
Inclusion	Studies meeting all eligibility criteria will be included in the final synthesis	Use of the PRISMA 2020 flow diagram to document the process transparently. All steps recorded and justified

All the references retrieved through the final search will be imported into reference management software (EndNote) and deduplicated. Screening will be supported by Rayyan, a web-based tool that streamlines the screening process. Titles and abstracts will be assessed for eligibility, followed by full-text review via predefined inclusion and exclusion criteria, guided by the SPIDER framework [40].

To minimise bias and enhance rigour, all records will be screened by at least two reviewers. The lead reviewer and other review team members will share the screening workload. Each study will be independently screened by two team members, and any discrepancies will be resolved through discussion during regular review meetings. Where consensus cannot be reached, the remaining reviewers will be consulted to provide additional perspectives.

**Fig. 1:**
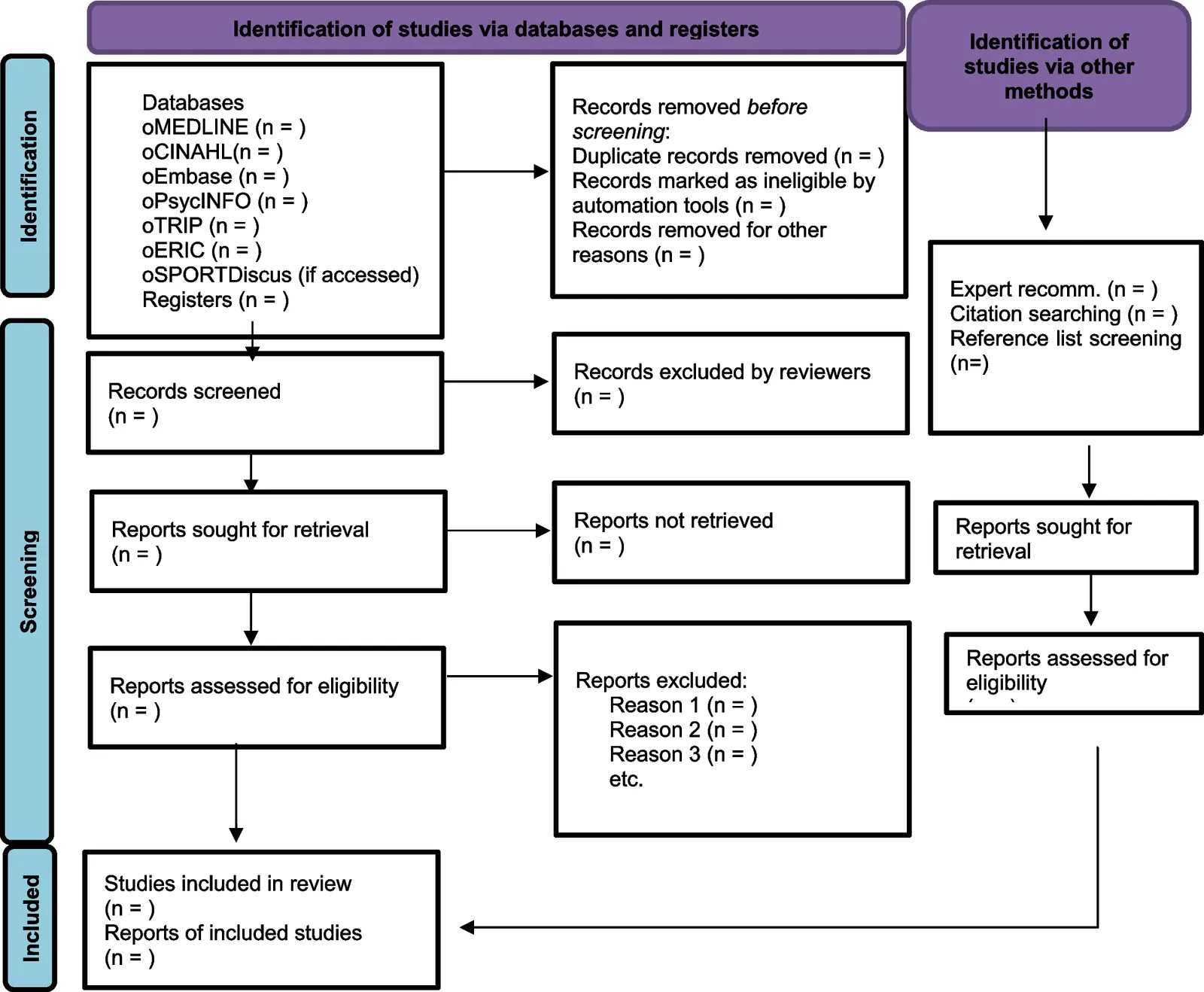
PRSMA flow diagram illustrating the study selection process (Page et al., 2021)

The appraisal results are summarised in tabular form and inform the synthesis process and the application of GRADE-CERQual [45]. The process will follow the PRISMA 2020 guidance, with all stages documented in a flow diagram (see Fig. 1) (Page et al., 2021). The PRISMA-S checklist will also be used to support transparent reporting of the search strategy [46].

#### Assessment of methodological quality and critical appraisal

A robust critical appraisal process will be implemented to maintain the integrity of the review and ensure that the evidence base reflects methodologically sound and credible research.

#### Appraisal tools and process

All eligible qualitative and mixed-methods studies will undergo critical appraisal using the JBI Critical Appraisal Checklist for Qualitative Research. [38]. Rather than assessing risk of bias in the conventional quantitative sense, the JBI checklist evaluates the methodological congruity and overall trustworthiness of qualitative research. This includes the alignment between the research methodology, research question, methods of data collection and analysis, analysis, interpretation of findings, researcher reflexivity, representation of participants’ voices, and ethical considerations.

Consistent with the guidance of Flemming and Noyes [47], appraisal outcomes will inform synthesis and assessment of confidence in the review findings rather than determine inclusion. Studies will not be excluded solely because of methodological limitations, recognising that qualitative research with minor methodological weaknesses may still provide valuable conceptual insights. Each study will be appraised independently by two reviewers, with discrepancies resolved through discussion with the wider review team. Appraisal findings will inform the assessment of methodological limitations within the GRADE-CERQual approach [45].

#### Data extraction and quality assessment

The data extraction sheet will be piloted on the first five eligible studies identified and reviewed to ensure that it is fit for purpose [38].

#### Data extraction process

Qualitative data will be extracted from each included paper. For mixed-methods studies, only the qualitative findings relevant to the review question will be extracted and synthesised. Where qualitative findings cannot be distinguished from quantitative results, the study will not contribute to the qualitative synthesis. The extracted information will include detailed characteristics of the study populations, context, culture, geographical setting, study design, and the phenomena of interest relevant to the review question and objectives. Key findings and their supporting illustrations (e.g. participant quotations) will also be extracted and assigned a level of credibility in line with JBI guidance [38].

The structured data extraction form will include the following elements (see Table 4 for an overview):

- Study characteristics: Author(s), year, country, setting, study aims, retrieval method, study title, and journal (if applicable).
- Participant characteristics: Description of participants (e.g. teachers, PE teachers, teaching assistants, coaches, or other school staff).
- Key findings and themes: Reported barriers, enablers, attitudes, beliefs, and other relevant factors.
- Methodological details: Type of qualitative study, data collection methods (e.g. interviews, focus groups).
- Methodological quality: Assessment using the JBI Critical Appraisal Checklist for Qualitative Research
- Ethical considerations: As reported by the authors.

**Table 4:** Data extraction table (aligned with SPIDER)

SPIDER element	Data item	Definition/notes
Sample	Study population details	Description of participants (e.g. PE teachers, general teachers, school staff), number of participants, teaching role, years of experience, training in diabetes care
	Study setting	Type of school (e.g. primary, secondary), location (e.g. urban/rural), country
	Recruitment method	How participants were recruited (e.g. purposive sampling, convenience sampling)
Phenomenon of interest	Perceptions, beliefs, attitudes, knowledge and experiences related to physical activity in CYP with T1D	Key concepts explored in relation to teachers’ understanding, support, and concerns regarding physical activity for children with T1D
Design	Data collection methods	How data were collected (e.g. interviews, focus groups, qualitative surveys)
Evaluation	Key findings	Main themes and subthemes identified, including illustrative participant quotes where available
	Interpretation and conclusions	Authors’ interpretations of the findings and implications for practice or policy
Research type	Type of qualitative research	Methodological approach (e.g. grounded theory, thematic analysis, interpretative phenomenological analysis)
Other	Funding source/conflicts of interest	Any declared funding or potential conflicts of interest
	Data analysis approach	Description of how data were analysed, coding methods, theme development, use of software if applicable
	Methodological quality	Methodological congruity and trustworthiness of the included studies will be appraised using the JBI Critical Appraisal Checklist for Qualitative Research
	Ethical considerations	No ethical approval required as this is a review of published studies. The review will follow ethical standards for secondary research, including accurate reporting, proper citation, and transparency

In addition, any pre-planned data assumptions and simplifications made during the synthesis process will be documented and justified. These may include decisions such as merging conceptually similar findings across studies or standardising terminology to enable comparison. See Table 5 for a summary of these assumptions and simplifications.

**Table 5:** Pre-planned data assumptions and simplifications

Assumption/simplification	Rationale
“Exercise” and “physical activity” will be treated as equivalent unless clearly distinguished	These terms are often used interchangeably in the literature; unless defined separately, they will be interpreted broadly
Where mixed participant groups are included, only data explicitly attributed to teachers will be extracted	To maintain focus on the target population (teachers); ambiguous quotes will be noted but not included in synthesis
If participant role or school type is not stated but can be reasonably inferred, this will be documented as an assumption	Allows inclusion of studies with minor gaps in reporting while maintaining transparency
If data collection or analysis methods are unclear, authors will be contacted. If no response, study will still be included but noted	To allow for comprehensive inclusion without compromising reporting rigour

#### Quality assessment

JBI meta-aggregation and GRADE-CERQual serve complementary purposes within this review. JBI meta-aggregation provides the methodological framework for synthesising qualitative findings, whereas GRADE-CERQual will be applied after the synthesis to assess the level of confidence that can be placed in each synthesised review finding.

Following completion of the meta-aggregation, confidence in each synthesised review finding will be assessed using the GRADE-CERQual approach [45]. CERQual evaluates confidence across four components:

- Methodological limitations—informed by the JBI Critical Appraisal Checklist for Qualitative Research;
- Coherence—the extent to which the review finding is well grounded in and consistent with the data from the contributing studies;
- Adequacy of data—the richness and quantity of the data supporting the review finding;
- Relevance—the applicability of the contributing studies to the review question.

The combined assessment across all four CERQual components will inform the overall confidence rating (high, moderate, low, or very low) assigned to each synthesised review finding.

#### Findings synthesis and reporting

This qualitative SR will employ the JBI meta-aggregation approach for synthesising findings. The synthesis process involves three steps: (1) extracting findings from studies, including supporting illustrations (e.g. participant quotes); (2) grouping findings by shared meanings; and (3) aggregating categories into synthesised statements that reflect the collective data. This approach preserves the original intent while making findings relevant for policy and practice [38].

The findings will be presented narratively, accompanied by summary tables outlining the study characteristics, quality assessments, and synthesised results.

#### Interpretation of the findings

The synthesised findings will be interpreted in relation to the review's objectives and within the broader context of the literature, educational policy, and practices concerning PA in CYP with T1D. Emphasis will be placed on implications for training, guidance, and support for school staff, especially PE teachers.

Findings will be synthesised using the JBI meta-aggregation approach. This involves three stages: (1) extraction of findings and supporting illustrations from the included studies; (2) grouping findings with similar meaning into categories; and (3) synthesising categories into comprehensive statements that preserve the meaning of the original studies while informing policy and practice [38].

This protocol has been developed in accordance with the PRISMA-P 2015 Statement to ensure clarity and transparency in reporting [48]. A completed PRISMA-P 2015 checklist is submitted as a supplementary file (Additional file 1).

#### Ethical considerations

As this review involves no primary data collection, formal ethical approval is not needed. However, ethics will be considered throughout the process and assessed within the included studies.

The JBI Critical Appraisal Checklist for Qualitative Research [38] will be used to evaluate ethical conduct, including approval, consent, and sensitivity to participants.

Following Flemming and Noyes [47], studies will not be excluded solely owing to ethical shortcomings, but concerns will be documented and reflected in the synthesis and GRADE-CERQual assessment [45].

The review will uphold ethical standards through transparency, minimise bias, acknowledge reflexivity, and respectfully represent vulnerable populations such as children and young people with long-term conditions.

### Outcomes

The primary outcomes of interest in this review are the perceptions, attitudes, knowledge, beliefs, experiences, and reported barriers or enablers held by school staff (e.g. teachers, PE teachers, teaching assistants) related to physical activity participation for CYP with T1D. Specifically, this includes:

- Understanding and awareness of physical activity guidelines and diabetes management within school settings
- Perceived challenges and concerns about supporting physical activity in CYP with T1D
- Facilitators and enablers that promote or support safe participation in physical activity

Additional outcomes include:

- Attitudes towards training, resources, and support needs for managing physical activity in CYP with T1D
- Experiences of implementing or adapting physical activity opportunities for CYP with T1D within schools
- Suggestions or recommendations from school staff for improving physical activity support

These outcomes will guide data extraction and synthesis to identify key themes that can inform interventions, training, and policy development.

## Discussion

This systematic review aims to synthesise qualitative evidence on teachers’ knowledge, perceptions, and experiences of supporting CYP with T1D in PA settings. By consolidating existing insights, this review aims to inform the development of school-based interventions, educator training, and policy guidance that promote safe and inclusive PA for this population.

While the review methodology is grounded in the JBI approach to qualitative evidence synthesis via meta-aggregation, several practical and operational considerations may impact the study. One such challenge involves the variability in how qualitative data are reported across studies. Differences in study context, reporting standards, and levels of detail provided in primary research may influence the ability to extract consistent and meaningful findings. To mitigate this, the team will use a piloted data extraction form and the structured JBI meta-aggregation process to ensure consistent identification, categorisation and synthesis of findings.

Another consideration is the diversity in educational systems, healthcare infrastructure, and cultural attitudes towards diabetes and physical activity internationally. This variation may affect the transferability of findings to the UK context, where this review is based. However, including international studies will enable the identification of broader themes and innovative practices that could inform UK policy and practice.

All included studies will be appraised via the JBI Critical Appraisal Checklist. However, the team acknowledges that quality assessment in qualitative reviews is inherently subjective and dependent on clear reporting in the original studies.

The engagement of a multidisciplinary review team, including experienced qualitative researchers and clinical specialists, will help ensure rigour, minimise bias, and support nuanced interpretation of the data. Screening and analysis will be conducted collaboratively, with regular discussions to resolve discrepancies and maintain consistency.

This protocol outlines a rigorous and transparent approach; however, limitations include the exclusion of non-English-language studies, which may result in language bias. The team also recognises that teachers’ perceptions may be influenced by systemic factors such as policy constraints, institutional culture, and access to training, which may not always be fully captured in primary research.

Ultimately, this review addresses an identified gap in the literature and addresses national priorities related to diabetes management, physical activity, and school inclusion. The findings will lay the foundation for the development of targeted, evidence-informed strategies to better equip educators and support the health and well-being of CYP with T1D.

## Supplementary Information

Supplementary Material 1: PRISMA checklist.

## Data Availability

No datasets were generated or analysed during the preparation of this protocol. The full search strategies will be provided as supplementary materials..Study selection and screening will be managed via Rayyan, and data coding and synthesis will be supported via NVivo software. Upon completion of the review, key data such as study characteristics, thematic findings, and GRADE-CERQual assessments will be made available as supplementary files in the final publication or in an open-access repository. Additional materials will be available from the corresponding author upon reasonable request. All changes to the protocol will be documented on PROSPERO.

## Abbreviations

T1D: Type 1 diabetes
CYP: Children and young people
PA: Physical activity
AACODS: Authority, Accuracy, Coverage, Objectivity, Date, Significance
CERQual: Confidence in the Evidence from Reviews of Qualitative Research
ERIC: Education Resources Information Center
JBI: Joanna Briggs Institute
NVivo: Qualitative Data Analysis Software
PRISMA: Preferred Reporting Items for Systematic Reviews and Meta-Analyses
PRESS: Peer Review of Electronic Search Strategies
SPIDER: Sample, Phenomenon of interest, Design, evaluation, Research type
